# Emerging Infectious Diseases in Free-Ranging Wildlife–Australian Zoo Based Wildlife Hospitals Contribute to National Surveillance

**DOI:** 10.1371/journal.pone.0095127

**Published:** 2014-05-01

**Authors:** Keren Cox-Witton, Andrea Reiss, Rupert Woods, Victoria Grillo, Rupert T. Baker, David J. Blyde, Wayne Boardman, Stephen Cutter, Claude Lacasse, Helen McCracken, Michael Pyne, Ian Smith, Simone Vitali, Larry Vogelnest, Dion Wedd, Martin Phillips, Chris Bunn, Lyndel Post

**Affiliations:** 1 Australian Wildlife Health Network, Mosman, New South Wales, Australia; 2 Zoo and Aquarium Association Australasia, Mosman, New South Wales, Australia; 3 Healesville Sanctuary, Zoos Victoria, Healesville, Victoria, Australia; 4 Sea World, Gold Coast, Queensland, Australia; 5 Adelaide Zoo, Zoos South Australia, Adelaide, South Australia, Australia; 6 Territory Wildlife Park, Berry Springs, Northern Territory, Australia; 7 Australia Zoo Wildlife Hospital, Beerwah, Queensland, Australia; 8 Melbourne Zoo, Zoos Victoria, Parkville, Victoria, Australia; 9 Currumbin Wildlife Sanctuary, Currumbin, Queensland, Australia; 10 Perth Zoo, South Perth, Western Australia, Australia; 11 Taronga Zoo, Taronga Conservation Society Australia, Mosman, New South Wales, Australia; 12 Australian Government Department of Agriculture, Canberra, Australian Capital Territory, Australia; The University of Hong Kong, Hong Kong

## Abstract

Emerging infectious diseases are increasingly originating from wildlife. Many of these diseases have significant impacts on human health, domestic animal health, and biodiversity. Surveillance is the key to early detection of emerging diseases. A zoo based wildlife disease surveillance program developed in Australia incorporates disease information from free-ranging wildlife into the existing national wildlife health information system. This program uses a collaborative approach and provides a strong model for a disease surveillance program for free-ranging wildlife that enhances the national capacity for early detection of emerging diseases.

## Introduction

Emerging infectious diseases are increasingly originating from wildlife, due in part to increasing urbanisation, globalised trade, habitat loss and other environmental changes. This is a real trend that cannot be fully explained by an increase in detection through improved surveillance, recognition, diagnosis or reporting [1], [2], [3], [4], [5]. Many of these diseases have significant impacts on human health, domestic animal health, wildlife health and biodiversity.

Zoonoses represent a rising threat to global health [5], [6]. Recent examples of emerging infectious diseases in humans with a wildlife origin include severe acute respiratory syndrome (SARS), Nipah virus and Ebola virus. Wildlife can act as a source and reservoir of diseases of domestic livestock such as bovine tuberculosis and avian influenza, and can result in significant economic losses [7], [8], [9]. Emerging diseases may also directly threaten wildlife health and biodiversity, as demonstrated in recent years by the emergence of white nose syndrome, Tasmanian devil facial tumour disease (DFTD) and chytridiomycosis [10], [11], [12], [13]. In Australia a number of diseases have emerged over the last 15 years with confirmed or suspected involvement of wildlife. Many of these diseases have had significant impacts on biodiversity, human health and domestic animal health, including chytrid fungus, DFTD, Australian bat lyssavirus (ABLV), Menangle virus, Japanese encephalitis and Hendra virus [14], [15], [16].

With the growing understanding of the importance of wildlife as a source or reservoir of emerging diseases, there is increased recognition of the need for disease surveillance in free-ranging wildlife. There are however inherent difficulties in conducting effective wildlife disease surveillance. Many wildlife disease events go unrecognised due to remote locations and a lack of obviously ill individuals or carcasses. Further challenges include a lack of validated diagnostic tests and laboratory capacity for the investigation of wildlife diseases, under-developed surveillance networks, difficulties in determining key parameters such as prevalence for diseases in wildlife populations, and lack of accurate ecological data on population size and density [17], [18]. Collection and validation of wildlife disease data can be challenging due to lack of funding, the ‘anecdotal’ nature of some reports, and the need to integrate data from disparate sources [16].

Utilising existing systems to establish a coordinated approach is an effective and efficient mechanism to overcome some of these difficulties, where they relate to reporting and data collection. This approach can be strengthened by a functional network that facilitates communication and information flow between those engaged at all levels in surveillance, diagnosis and management of wildlife disease. Surveillance information collected in this way may contribute to the early detection of new or emerging diseases [16], [19]. This paper describes a zoo based wildlife disease surveillance program, as an example of how such a system can assist in managing some of the issues associated with disease surveillance in free-ranging wildlife.

In Australia, the national animal health system is supported by a co-ordinated general wildlife health surveillance system. The primary responsibility for gathering animal health data, including wildlife disease data, rests with state and territory government agencies [20]. The Australian Wildlife Health Network (AWHN) is a national network of government and private stakeholders with an interest in wildlife health that receives core funding from the Australian Government Department of Agriculture. The AWHN is charged with collation and management of national wildlife surveillance data, and works within a ‘One Health’ framework by encouraging collaboration on wildlife health issues and investigations across human health, animal health and environmental sectors [21]. The AWHN manages wildlife health data through a national web-based database known as eWHIS (the ‘electronic Wildlife Health Information System’). A key component of the wildlife health surveillance system are the ‘wildlife coordinators’, with a government representative in each of Australia’s states and territories. Wildlife coordinators manage wildlife disease investigations in their jurisdiction and report data into eWHIS. State, territory and commonwealth agriculture, environment and human health agencies, universities, private veterinary practices and zoos all contribute to Australia’s coordinated wildlife health surveillance system. The zoo based wildlife disease surveillance program was developed to formally incorporate disease information from free-ranging wildlife presented to Australian zoos into this existing national wildlife health information system.

Zoos are well suited to participation in surveillance efforts, as many zoos conduct active disease surveillance of collection animals as part of their routine preventative medicine programs, maintain serum and tissue banks and detailed medical records, and have staff with technical expertise in wildlife health [22], [23], [24]. The Zoo Animal Health Network in the USA, for example, is a collaborative program with the United States Department of Agriculture that is involved in early disease detection and outbreak response programs [25], [26], [27], [28]. The value of zoos for surveillance was demonstrated in 1999 when investigation of wild bird mortalities by veterinarians at New York City’s Bronx Zoo led to the diagnosis of the first known occurrence of West Nile virus (WNV) in the western hemisphere, a disease with significant human and animal health impacts [22], [23].

Typically, however, zoo surveillance has largely focused on captive animals within zoo collections. In Australia, wildlife hospitals operated by the major zoos also treat a significant caseload of free-ranging and rehabilitation wildlife. A survey in 2008 found that 15 Australian zoos treated over 14,000 wildlife cases each year in their wildlife hospitals [29] and admissions to these hospitals appear to be increasing over time. As well as providing expertise in veterinary care, these hospitals have strong links to a network of wildlife rehabilitation, conservation, research and welfare organisations in their region.

The zoo based wildlife disease surveillance program was developed in recognition of the strong capacity and potential for wildlife hospitals at Australian zoos to contribute to national and international wildlife disease surveillance. The program aimed to integrate zoo based wildlife hospitals into Australia’s animal health surveillance system. This paper describes the program and reviews the outcomes in the context of wildlife diseases that impact on human health, livestock health, trade and biodiversity.

## Materials and Methods

### Planning

In 2009 the Zoo Animal Health Reference Group [30] held a workshop to identify the role that Australian zoos could play in biosecurity, and surveillance was identified as a key area where a contribution could be made. A zoo based wildlife disease surveillance program was proposed and a collaborative project was subsequently developed between the AWHN and the Zoo and Aquarium Association Australasia (ZAA). The ZAA, with over 80 institutional members, is the peak body representing the zoo and aquarium industry in Australia and New Zealand. The AWHN and the ZAA worked with the Zoo Animal Health Reference Group and the senior veterinarians from the participating zoos to develop the scope and methodology for a pilot project to evaluate the potential of a zoo based surveillance program. The aim of the pilot project was to trial the integration of free-ranging wildlife disease information from zoo based wildlife hospitals into the national wildlife health information system. An additional objective was to strengthen and improve communication and the flow of information between zoo veterinarians and relevant government agencies.

**Figure 1:**
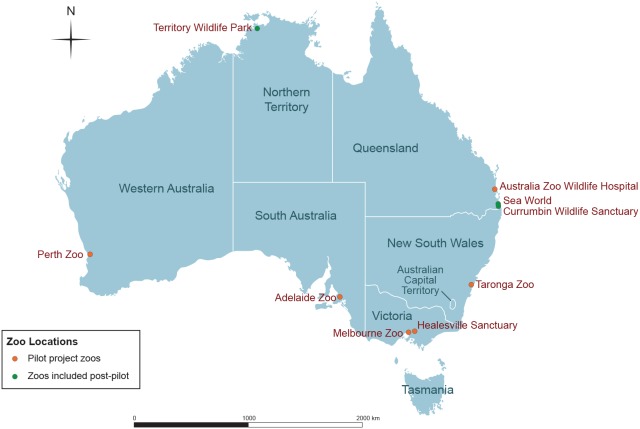
Geographic location of the zoos participating in the surveillance program.

Six major Australian zoos were selected to participate in the pilot project, each with a well-established and resourced on-site veterinary hospital treating free-ranging and rehabilitation wildlife and a permanent staff of experienced zoo and wildlife veterinarians. The six participating zoos are located in five Australian states: Adelaide Zoo in South Australia, Australia Zoo Wildlife Hospital in Queensland, Healesville Sanctuary and Melbourne Zoo in Victoria, Perth Zoo in Western Australia and Taronga Zoo in New South Wales (Figure 1). A formal survey of these zoos was conducted to gather baseline information and assist in planning for the pilot project. Data were collected on the number and taxonomic breakdown of wildlife cases seen by each of the zoo veterinary hospitals over a 12-month period during 2009 to 2010 (Table 1).

**Table 1:** Indicative numbers of free-ranging wildlife cases seen by veterinary hospitals at six major Australian zoos over a 12 month period during 2008/2009.

	Native species*				Feral species*	
ZOO	Mammals	Birds	Reptiles	Amphibia	All taxa	TOTAL
**Australia Zoo Wildlife Hospital**	2,579 (38%)	2,835 (42%)	1,126 (17%)	49 (0.7%)	197 (3%)	**6,786**
**Healesville Sanctuary**	567 (37%)	851 (56%)	95 (6%)	14 (0.9%)	*	**1,527**
**Taronga Zoo**	276 (39%)	341 (48%)	92 (13%)	5 (0.7%)	*	**714**
**Perth Zoo**	75 (12%)	328 (53%)	188 (31%)	8 (1%)	15 (2%)	**614**
**Melbourne Zoo**	135 (37%)	135 (37%)	76 (21%)	23 (6%)	*	**369**
**Adelaide Zoo**	85 (39%)	100 (46%)	8 (4%)	2 (0.9%)	18 (8%)	**213**
**TOTAL**	**3,717 (36%)**	**4,590 (45%)**	**1,585 (16%)**	**101 (1%)**	**230 (2%)**	**10,229**

*Data from three zoos did not differentiate feral from native species; for these zoos, feral animal cases are included with native species numbers.

### Operation

The pilot project commenced in November 2010 and finished in October 2011. During this time an agreed data set was collected from free-ranging and rehabilitation wildlife cases seen by the participating zoo veterinary hospitals. The scope of the pilot project did not include data from zoo collection animals and focused on the reporting of existing work, rather than expansion of disease investigations. Reporting into the national wildlife health information system in the pilot project was limited to selected disease event categories (Table 2), which had previously been established as a high priority for wildlife surveillance in Australia and aligned with data being reported from other sources. These categories are designed to collect wildlife disease information of potential importance to human health, livestock health, trade and biodiversity. While the priority for data collection was positive results, reporting of negative results was also encouraged, particularly where a specific disease was excluded that is a locally, nationally, or internationally notifiable or reportable disease. The ‘interesting or unusual’ category was designed to capture unusual events or findings that could indicate an emerging disease, syndrome or trend. Examples of disease events that could be reported in this category are significant clusters or patterns of disease, unexpected morbidities or mortalities, toxicity events, marine wildlife strandings, and cases with possible linkages to international events or drivers. Cases for reporting were not confined to those where a necropsy or laboratory test had been conducted. Participants were encouraged to report a range of cases using different diagnostic tools, including where the diagnosis was based solely on clinical examination.

**Table 2:** Categories for selection of wildlife disease events for reporting into eWHIS.

Category
World Organisation for Animal Health (OIE) Listed diseases
Bat viral diseases
Mass mortalities
Arboviral diseases
Salmonella cases
‘Interesting or unusual’ cases

Cases were reported into the national wildlife health surveillance system via a web-enabled database, the ‘electronic Wildlife Health Information System’ (eWHIS). After initial training sessions provided by the AWHN, the zoo veterinarians entered data directly into the eWHIS database on a monthly basis for the duration of the pilot project, with ongoing training and support provided as needed. Fields captured included: event dates, event location, event type (e.g. individual, outbreak, monitoring), event category (see Table 2), species, number (affected and dead), state of captivity, presenting syndrome, diagnosis (one or multiple), laboratory test details and confidentiality level. The data entered into eWHIS were reviewed and moderated by the AWHN.

Participating zoo veterinarians were given the opportunity to discuss interesting disease events and operational aspects of the pilot project at regular teleconferences. All zoo participants were encouraged to engage with their state or territory agriculture agency via the wildlife coordinator, however this did not replace or bypass the legislated reporting of notifiable animal diseases through appropriate channels.

### Evaluation

An independent review was conducted at the end of the pilot project by an internationally-recognised consulting company with expertise and experience in epidemiology and wildlife disease surveillance. Their evaluation of the project included an assessment of the value of the surveillance data and the potential for the project to deliver benefits to stakeholders, including the Australian commonwealth, state and territory governments. The sustainability of the system was also assessed. The evaluation process included an online stakeholder survey, interviews with the project coordinators and analysis of collected data.

## Results

The preliminary survey indicated that the six zoos treated over 10,000 wildlife cases in a year (Table 1). All six selected zoos agreed to participate in the pilot project and the zoo veterinarians commenced entering data directly into the eWHIS database from November 2010. Sixteen zoo veterinarians participated for some or all of the pilot project period. A total of 211 events that occurred during the 12-month pilot project were reported into eWHIS by the participating zoos. This represented almost a third of all cases submitted to eWHIS during that period from all sources including state and territory departments of agriculture and human health, university researchers and private veterinary practitioners. A small subset of the cases presented to the zoo veterinary hospitals met the agreed criteria for data entry into eWHIS (Table 2). This subset was between 1 and 8% of all cases for individual zoos and approximately 2% for the six zoos overall. Examples of disease events reported for each of the categories are provided in Table 3.

**Table 3:** Examples of disease events captured for each reporting category (see Table 2).

Reporting Category	Examples
**OIE Listed diseases** *	• Avian chlamydiosis (*Chlamydophila psittaci)*
	• Botulism
	• Psittacine circoviral (beak and feather) disease
	• Toxoplasmosis
	• Trichomoniasis
**Bat viral diseases**	• Australian bat lyssavirus
**Mass mortalities**	• Six Carnaby’s black-cockatoos (*Calyptorhynchus latirostris*) found dead in a similar location over a two-week period
	• Twenty-one rainbow lorikeets (*Trichoglossus haematodus*) and scaly-breasted lorikeets (*Trichoglossus chlorolepidotus*) with neurological signs over a period of a month
**Arboviral diseases**	• None reported
**Salmonella cases**	Salmonella cultured from:
	• Green turtle (*Chelonia mydas*) – corneal abscess
	• Australian Raven (*Corvus coronoides*) with neurological signs – muscle
	• Two hand-raised eastern grey kangaroos (*Macropus giganteus giganteus*) with diarrhoea and anorexia – faeces
	• Hand-raised koala (*Phascolarctos cinereus*) joey with neurological signs and septicaemia - caecum, blood and liver
**‘Interesting or unusual’ cases**	• Fourteen cases of neoplasia in a variety of species including yellow-bellied glider (*Petaurus australis*), New Zealand fur seal (*Arctocephalus forsteri*), koala (*Phascolarctos cinereus*), wedge-tailed eagle (*Aquila audax*), laughing kookaburra (*Dacelo novaeguineae*)
	• Australian fur seal (*Arctocephalus pusillus doriferus*) with acute suppurative meningitis; heavy growth of *Arcanobacterium*
	• Multisystemic lymphoproliferative disease in a wedge-tailed eagle (*Aquila audax*)
	• Green turtle (*Chelonia mydas*) with fibropapillomatous lesions on flippers
	• Australian raven (*Corvus coronoides*) with non-suppurative encephalitis; flavivirus, avian influenza and Newcastle disease excluded

*Includes ‘non-listed’ pathogens and agents of wildlife [49].

A wide range of wildlife species was represented by the data collected during the pilot project. Accurate taxonomic identification of the animals under investigation was possible due to the expertise of the participating zoo veterinarians. The 211 disease events reported for the period from November 2010 to October 2011 covered 52 different species from 31 families and included birds (12 orders), turtles, marsupials, monotremes, marine mammals and bats (Table 4). The pilot project increased the overall species coverage of the data collected in eWHIS, with 18 species (9 bird, 7 mammal and 2 reptile species) reported through the pilot project that were not reported from other sources during the same period. A number of events reported through the pilot project came from geographic areas not represented by other sources.

**Table 4:** Cases* for November 2010– October 2011 reported through the pilot project, by taxonomic group.

Taxonomic group	
**A. ALL CASES**	**No. of cases (%)**
**Birds**	109 (52%)
**Mammals**	79 (37%)
**Reptiles**	23 (11%)
**Total**	**211**
**B. MAMMALS**	**No. of cases (% of mammal cases)**
**Non-macropod marsupial**	34 (43%)
**Bat** +	28 (35%)
**Macropod**	10 (13%)
**Marine mammal**	4 (5%)
**Monotreme**	2 (3%)
**Other mammal**	1 (1%)
**Total**	**79**

*A case may involve single or multiple animals.

+ The majority of bat cases were submitted for exclusion testing for Australian bat lyssavirus (ABLV).

The project captured data on diseases with potential human health implications, including confirmed or highly suspicious cases of salmonellosis, avian chlamydiosis (*Chlamydophila psittaci)*, Australian bat lyssavirus in bats, mycobacteriosis (unspeciated) in a koala (*Phascolarctos cinereus*), and cryptosporidiosis in a hand-raised macropod. The cases of salmonellosis occurred in a variety of birds, marsupials and reptiles, and typing of these isolates contributed to the National Enteric Pathogens Surveillance Scheme [31]. Multiple cases of neurological signs in tawny frogmouths (*Podargus strigoides*) in urban areas of Sydney were of interest as this species has been suggested as a sentinel for the emerging zoonosis angiostrongylosis [32], [33].

Of the records entered in eWHIS through the pilot project, 73% were categorised by the submitter as ‘interesting or unusual’, a grouping designed to capture information on possible emerging syndromes and trends. As an example, 14 cases of neoplasia were reported. Cancers have been recognised as emerging diseases of wildlife with potentially serious impacts, including Tasmanian devil facial tumour disease [11] and fibropapillomatosis of green turtles (*Chelonia mydas*) [34]. Additionally, cancer clusters in wildlife due to environmental causes such as chemical contamination can act as sentinels for risk to human health [35], [36]. Cases were also reported of recognised syndromes where the cause has not been fully identified, such as non-suppurative encephalitis in corvids and paralysis in rainbow lorikeets (*Trichoglossus haematodus*). This information could contribute to a better understanding of syndromes with unknown aetiology.

Cases in threatened species were reported, including the endangered Carnaby’s black-cockatoo (*Calyptorhynchus latirostris*) and loggerhead turtle (*Caretta caretta*) and a number of vulnerable species (Table 5) [37], some of which were not represented in data captured from other sources for the same period. Data were collected on cases of psittacine circoviral (beak and feather) disease, which is listed as a key threatening process in endangered psittacine species under the *Environment Protection and Biodiversity Conservation (EPBC) Act 1999* [38]. Also reported was a diabetes syndrome affecting koalas in care that could impact on the rehabilitation success of koalas in Queensland, a species now listed as vulnerable under the *EPBC Act 1999* [37]. The first two confirmed clinical cases of chlamydiosis in koalas in South Australia were reported to the AWHN through the pilot project [39]. The South Australian koala population was thought to be free of *Chlamydia* [40], so these reports may be an indicator of an emerging disease in the South Australian koala population.

**Table 5:** Threatened species for which data was captured through the pilot project.

Species	EPBC Act Listing Status
Carnaby’s black-cockatoo(*Calyptorhynchus latirostris*)	Endangered
Loggerhead turtle(*Caretta caretta*)	Endangered
Chuditch or Western quoll(*Dasyurus geoffroii*)	Vulnerable
Flatback turtle(*Natator depressus*)	Vulnerable
Green turtle (*Chelonia mydas)*	Vulnerable
Grey-headed flying fox(*Pteropus poliocephalus*)	Vulnerable
Hawksbill turtle(*Eretmochelys imbricata*)	Vulnerable
Koala *(Phascolarctos cinereus*)*	Vulnerable
Quokka (*Setonix brachyurus*)	Vulnerable
Sub-Antarctic fur seal(*Arctocephalus tropicalis*)	Vulnerable

*The koala (combined populations of Queensland, New South Wales and the Australian Capital Territory) was listed as vulnerable in May 2012.

The project provided a framework for improved data capture for monitoring programs. For example, the AWHN holds responsibility for collating, moderating and maintaining a national dataset of bats tested for ABLV, and the pilot project resulted in the capture of more detailed information on the history and clinical signs of bats for this dataset.

The project framework assisted the management of a disease outbreak in 2011. A strain of avian paramyxovirus 1 (APMV1) not previously reported in Australia was detected in hobby pigeons in the Melbourne area in Victoria, and the virus was subsequently detected in free-living feral rock doves (*Columba livia*) and a spotted turtle dove (*Streptopelia chinensis*), and in a native collared sparrow hawk (*Accipiter cirrocephalus*) [41], [42]. The project provided a mechanism to update zoo veterinarians about the outbreak, highlighted the possible involvement of native pigeons and raptors, and most likely resulted in increased submission of free-ranging sick and dead birds to the Victorian Department of Primary Industries for testing. A number of notifications of other disease events and outbreaks of relevance to wildlife were disseminated through the project, including a cluster of Hendra virus cases in horses in New South Wales and Queensland [43], and neurological disease in horses due to arboviruses in New South Wales [44] in 2011.

Information reported into eWHIS by the participants contributed to Australia’s reports to the World Organisation for Animal Health (OIE). Australia, as a contributor to the OIE, regularly reports on the country’s animal health status, which is important to ensure that Australia’s health status for animals and animal products is well recognised internationally [45].

### Evaluation

The independent review found that the pilot project increased the volume of cases and expanded the sources of data being entered into the national database [46]. According to the review, the project resulted in increased geographic and taxonomic coverage of the wildlife population, with data collected from additional ‘catchment’ areas and an increased species distribution, as well as a wider range of presenting syndromes and reporting reasons. The review concluded from these outcomes that the pilot project enhanced the capacity of the national wildlife health information system for early detection of disease and improved the sensitivity for demonstration of freedom from disease.

The survey of zoo participants found that most considered their institution had benefited from the pilot project. Participants reported that the project provided additional focus for the zoos to investigate wildlife diseases; resulted in better recognition of their contribution to wildlife health; and improved collaboration, connection and communication with other institutions and organisations. It also contributed to a better understanding of the wider context of wildlife disease events, and assisted in identifying patterns in these events by providing a forum to share information on similar syndromes from different locations. The majority of participants agreed that participation in the project increased their awareness and understanding of diseases of national concern. The review identified some limitations of the program, including the clustering of cases around major population centres, and the collection of only a small proportion of the total caseload of the participating zoo wildlife hospitals.

The reviewers concluded that there was value in the project to both the stakeholders and the participants, and that it was sustainable. They recommended the program be continued and expanded to include more zoos in order to increase the coverage and volume of data collected and to build on the improved capacity for early detection of wildlife disease. Factors recommended for consideration in the selection of additional zoos included geographic location and the ‘catchment’ area of wildlife covered by the zoo, veterinary presence, caseload, nature of cases, and availability of resources for data entry.

### Outcomes

Based on the success of the pilot project and the recommendations of the independent review, the zoo based wildlife disease surveillance program has continued. Each of the participating zoos has remained with the program, which has expanded to incorporate three additional zoos with the aim of increasing both the geographic and species range. These zoos are Currumbin Wildlife Sanctuary and Sea World in Queensland, and Territory Wildlife Park in the Northern Territory (Figure 1). This brings the total number of free-ranging wildlife cases seen by the nine participating zoos to around 17,000 cases each year. A total of 25 zoo wildlife hospital staff have directly participated in the program since its inception.

## Discussion

Animal health surveillance is the key to early detection and management of emerging diseases. The need to include free-ranging wildlife populations in animal health surveillance programs is increasingly recognised in Australia and globally [1], [5], [14], however effective disease surveillance in free-ranging wildlife populations presents many challenges. In Australia, as in many countries, there is an established system for investigating wildlife disease events and reporting them into the national system, however a considerable number of wildlife cases are inevitably seen outside of this system. A significant caseload of free-ranging wildlife is presented for treatment to Australian zoo based veterinary hospitals by members of the public, wildlife carers and park rangers, or are referred by state and territory government agencies, and the cost of providing this service is mostly covered by the zoos’ operating budgets [29].

Australian zoo based hospitals are recognised as one of the chief sources of information on wildlife health and are well placed to participate in wildlife disease surveillance as these zoos have veterinary staff with expertise in wildlife health, are well organised and represented by their peak body, the Zoo and Aquarium Association, and have an existing framework of communication and collaboration. Zoos also have strong linkages with a broad network of wildlife rehabilitators, wildlife researchers, conservation organisations and environmental officers in their districts. For these reasons, the existing framework for the national reporting of wildlife disease information was expanded to include zoo veterinarians working with free-ranging wildlife. A pilot project demonstrated that a zoo based surveillance program was able to capture useful information on disease in free-ranging wildlife that might otherwise not have been reported into the national system, or was reported earlier than would otherwise have occurred. The program has the ability to capture valuable information on diseases of humans and domestic animals originating from wildlife, diseases in threatened species and recognised syndromes of unknown aetiology.

Some limitations of the zoo surveillance pilot project were identified by the independent review and the authors. Geographic coverage of cases reported through the project was, as expected, clustered around the physical locations of the participating zoos, which are primarily in or near the major population centres in coastal areas of Australia. This reflects the inherent bias of general surveillance systems. Although primarily in coastal locations, the zoos are situated in a variety of geographic and climatic zones, and in both urban and rural settings. This source of surveillance information does not stand alone, but complements other sources of data. The program also allows clear identification of geographic areas where general surveillance is of lower intensity, which is valuable for planning and assessment of risk.

As described, the scope of the project resulted in the collection of only a small proportion of the total caseload of the zoo wildlife hospitals into the eWHIS database (1–8%). The majority of cases presenting to zoo wildlife hospitals involve orphaned animals and cases involving dog, cat or vehicular trauma. Most of these do not align with the categories for reporting, which are selected on the basis of nationally-agreed priorities for wildlife disease surveillance in Australia. Nonetheless a large volume of potentially valuable data is not captured through the program, and this aspect of data collection will be further investigated by the authors. There may also be cases that meet the selection criteria but are not being reported into eWHIS, as the decision on what to report rests with the submitter, however the AWHN provides training and ongoing guidance on case selection to minimise the loss of eligible data.

This program focuses on wildlife hospitals at zoos, however the caseload varies significantly between participating institutions and in some instances there are other organisations in the same region with a higher caseload, such as private veterinary clinics, and not-for-profit wildlife hospitals and rehabilitation centres, which are not yet formally integrated into the surveillance system. This program may be used as a model in future to integrate other types of organisations into the national wildlife health surveillance system.

The Australian zoo based wildlife disease surveillance program provides a model for an effective, low cost system that utilises existing capacity and routine activities to contribute to national and international surveillance efforts. The program generates information with the potential to assist earlier detection of emerging diseases and trends, as well as strengthening networks, improving communication and information flow, and building capacity in wildlife health professionals. These elements form the basis of a successful surveillance program. This program acknowledges the value of data where a range of diagnostic tools, including clinical assessment has been used. As a model, it demonstrates that meaningful surveillance can be conducted in a variety of circumstances, including those where laboratory capacity and financial resources are limited.

There is a recognition that successful surveillance relies on communication between stakeholders, including private practitioners and public officers [47]. There is a need for greater integration and linkage of animal - both wild and domestic - and human pathogen surveillance systems at the international and national level [48]. The need for a systematic approach to communication between the human and animal disease surveillance systems in Australia has been outlined [19]. A ‘One Health’ approach can result in increased interaction between professionals working in the veterinary, medical, wildlife and environmental spheres [14]. In an evaluation of the WNV surveillance program in the USA, an association was found between submission of samples by zoos for WNV testing and the level of communication between the zoos and the public health agency [24]. The authors concluded that a greater awareness of the importance of surveillance by zoos could result in better collaboration and detection of possible human health threats from animal disease events.

The AWHN maintains a ‘first alert’ framework based on a national network of wildlife health professionals that can be used to coordinate and disseminate information in an emergency or a significant disease event. This network receives regular notifications of disease alerts, requests for information and samples, and publication of significant articles, guidelines and policy documents. The pilot project demonstrated the potential of the program to widen this network and raise the level of awareness of emerging diseases and diseases of potential national importance. The collaborative framework of the program also encourages discussion on new and interesting events and patterns of disease across multiple locations, and facilitates sharing of samples for testing and research.

The program has resulted in improved communication and flow of information, and strengthened relationships between the zoo industry and government agencies, in particular the state and territory departments of agriculture. Linking with zoos provides an avenue for information gathering and dissemination, and an opportunity to utilise the expertise and resources within their extensive networks. The program has the potential to build the capacity of zoos to play a rapid and effective role in a disease emergency by integrating zoo veterinarians into the national biosecurity surveillance network.

## Conclusion

The science of understanding emerging infectious diseases with wildlife as part of their ecology has gained much attention over recent years, but it is often difficult to conduct meaningful surveillance in this area. The Australian zoo based wildlife disease surveillance program uses a collaborative approach involving government and the zoo industry, with a focus on collecting and reporting of wildlife disease events with potential impact on human health, livestock health and biodiversity. It provides a strong model for a disease surveillance program for free-ranging wildlife that could be adapted and utilised in other contexts. There is potential for expansion of the program to groups outside of zoo hospitals such as private veterinary practitioners from ‘sentinel’ hospitals with a high wildlife caseload, veterinary hospitals run by animal welfare organisations and universities involved in clinical wildlife work and research. Integration of these groups into the national wildlife health surveillance system has the potential to assist in the early detection of emerging diseases in Australia’s free-ranging wildlife population.
